# Multidrug-Resistant Tuberculosis and Its Association with Adrenal Insufficiency: Assessment with the Low-Dose ACTH Stimulation Test

**DOI:** 10.1155/2016/9051865

**Published:** 2016-02-23

**Authors:** René Rodríguez-Gutiérrez, Adrian Rendon, Maximiliano Barrera-Sánchez, Kevin Erick Gabriel Carlos-Reyna, Neri Alejandro Álvarez-Villalobos, Gloria González-Saldivar, José Gerardo González-González

**Affiliations:** ^1^Endocrinology Division, University Hospital “Dr. Jose E. Gonzalez”, Medical School, Autonomous University of Nuevo Leon, 64460 Monterrey, NL, Mexico; ^2^Department of Internal Medicine, University Hospital “Dr. Jose E. Gonzalez”, Medical School, Autonomous University of Nuevo Leon, 64460 Monterrey, NL, Mexico; ^3^Knowledge and Evaluation Research Unit in Endocrinology, Mayo Clinic, Rochester, MN 55905, USA; ^4^Division of Endocrinology, Diabetes, Metabolism & Nutrition, Department of Medicine, Mayo Clinic, Rochester, MN 55905, USA; ^5^Centro de Investigacion, Prevencion y Tratamiento de Infecciones Respiratorias (CIPTIR), Autonomous University of Nuevo Leon, 64460 Monterrey, NL, Mexico; ^6^Clinical Research Unit, Medical School, Autonomous University of Nuevo Leon, 64460 Monterrey, NL, Mexico

## Abstract

*Background*. Multidrug-resistant tuberculosis (MDR-TB) is a major public health care concern that affects the life of millions of people around the world. The association of tuberculosis and adrenal insufficiency is well known; however, it is thought to be less prevalent every time. A spike in TB incidence and a lack of evidence of this association in patients with MDR-TB call for reassessment of an illness (adrenal dysfunction) that if not diagnosed could seriously jeopardize patients' health.* Objective*. To determine the prevalence of adrenocortical insufficiency in patients with MDR-TB using the low-dose (1 *μ*g) ACTH stimulation test at baseline and at 6–12 months of follow-up after antituberculosis treatment and culture conversion.* Methods*. A total of 48 men or women, aged ≥18 years (HIV-negative patients diagnosed with pulmonary MDR-TB) were included in this prospective observational study. Blood samples for serum cortisol were taken at baseline and 30 and 60 minutes after 1 *μ*g ACTH stimulation at our tertiary level university hospital before and after antituberculosis treatment.* Results*. Forty-seven percent of subjects had primary MDR-TB; 43.8% had type 2 diabetes; none were HIV-positive. We found at enrollment 2 cases (4.2%) of adrenal insufficiency taking 500 nmol/L as the standard cutoff point value and 4 cases (8.3%) alternatively, using 550 nmol/L. After antituberculosis intensive phase drug-treatment and a negative mycobacterial culture (10.2 ± 3.6 months) adrenocortical function was restored in all cases.* Conclusions*. In patients with MDR-TB, using the low-dose ACTH stimulation test, a low prevalence of mild adrenal insufficiency was observed. After antituberculosis treatment adrenal function was restored in all cases. Given the increasing and worrying epidemic of MDR-TB these findings have important clinical implications that may help clinicians and patients make better decisions when deciding to test for adrenocortical dysfunction or treat insufficient stimulated cortisol levels in the setting of MDR-TB.

## 1. Introduction

In the past century, tuberculosis (TB) prevalence, worldwide, tended to plateau [1]. In recent decades, however, its incidence has increased and has become one of the major global public health care concerns [1, 2]. In fact, over two billion people are infected today by* Mycobacterium tuberculosis*, leading to ~5,000 deaths every day [3]. Interestingly, ~80% of the newly diagnosed cases occur in high-burdened countries, mainly in Asia and Africa [3]. These places are at increased risk from drug-resistant strains due to limited access to medical attention, delay in diagnosis, and chemotherapy initiation and as a result of low therapeutic adherence. Approximately half a million of multidrug-resistant tuberculosis (MDR-TB) cases are diagnosed per year; however, this number is too optimistic as less than 7% are reported to the World Health Organization [4]. Hence, MDR-TB and its comorbidities, including adrenocortical dysfunction, are expected to be faced more often.

Autoimmune adrenalitis is still referred to, however, as the most frequent etiology of primary adrenal insufficiency, under the notion that TB has been surpassed due to a decrease of its incidence [5–7]. This assumption needs to be reassessed in light of the global reemergence of TB and MDR-TB. Prevalence studies of adrenocortical dysfunction in patients with TB using the high-dose ACTH stimulation test have found a broad proportion going from zero to 54% [8–11]. Only one study has been carried out in patients with MDR-TB, in which, by using the high-dose adrenocorticotropin hormone (ACTH) test (250 *μ*g), a 49.5% prevalence of adrenocortical dysfunction was reported [11]. Recent studies, however, have shown that the low-dose ACTH stimulation test (1 *μ*g), a more physiologic dose, is more reliable test in detecting primary adrenocortical insufficiency [12, 13]. To our knowledge, no patients with MDR-TB have been evaluated by the low-dose ACTH stimulation test. It is expected that this complicated population may have an increased incidence of primary adrenocortical insufficiency among other factors due to the extended exposure to the mycobacteria and low adherence to therapy.

Accordingly, in this study, the low-dose ACTH stimulation test was used to determine the prevalence of adrenocortical insufficiency in patients with MDR-TB at enrollment and during follow-up 6 months later, after mycobacterial culture conversion. Secondary endpoints included the following: (1) assessing the differences between primary and secondary MDR-TB patients and adrenocortical dysfunction, (2) contrasting the prevalence of adrenocortical insufficiency at two different cortisol cutoff point values, and (3) identifying any clinical features related to an abnormal adrenal function.

## 2. Subjects and Methods

After obtaining approval from the research and ethics committee we began the study. Patients were recruited from the Research Center for Prevention and Treatment of Respiratory Infections (CIPTIR) of the University Hospital “Dr. José E. González”, Universidad Autónoma de Nuevo León. Each subject signed the informed consent form before being enrolled. A total of 48 men or women, aged ≥18 years, diagnosed with pulmonary MDR-TB were included. MDR-TB diagnosis was established on the basis of a disease caused by strains of* Mycobacterium tuberculosis* resistant to Isoniazid and Rifampicin [4]. Primary MDR-TB diagnosis was defined as drug-resistance in a case with no history of prior infection with treatment-sensitive TB strains [4]. Secondary drug-resistance refers to the development of resistance in patients with previously drug-susceptible TB [4]. A complete clinical history was collected from all participants. Emphasis was given to comorbidities, medication used, and clinical signs of adrenocortical failure or pulmonary TB. We excluded subjects with other endocrinopathies, HIV infection, glucocorticoid use in the last 12 months, and actual use of oral/nonoral contraceptives or drugs that may affect cortisol metabolism, transport, or the hypothalamo-pituitary-adrenal (HPA) axis.

### 2.1. Study Protocol

To perform the low-dose ACTH stimulation test, the solution was prepared from serial dilutions in normal saline from one vial of 250 *μ*g synthetic ACTH (Cortrosyn*™*, Amphastar Pharmaceuticals, Inc., Rancho Cucamonga, CA, USA), first to 50 *μ*g/mL, then to 5 *μ*g/mL, and finally to 1 *μ*g/mL concentration. All subjects underwent 1 *μ*g ACTH stimulation dose test. After overnight fasting, between 0800 h and 0900 h, a baseline blood sample was drawn for serum cortisol measurement. Subsequently, 1 *μ*g ACTH was injected as a bolus and flushed afterwards with 10 mL of normal saline. Blood samples for serum cortisol were taken at 30 and 60 minutes. A serum cortisol value equal to or greater than 500 nmol/L at 30 or 60 minutes after stimulation was defined as a normal glucocorticoid response [14]. An alternative cortisol cutoff value (550 nmol/L) was also examined [15]. This test was repeated, not before 6 months, once mycobacterial cultures were confirmed negative.

### 2.2. Measurements

Serum cortisol was measured using a commercial RIA kit (Elecsys 2010, Roche/Hitachi Diagnostics, Mannheim, Germany). Intra-assay variation coefficients were 1.6, 1.5, and 1.6% for low, medium, and high cortisol values, respectively. All samples were assessed twice.

### 2.3. Statistical Analysis

Descriptive statistical analysis was used for quantitative variables, measures of central tendency, and dispersion; all results are reported as mean ± standard deviation (SD) unless otherwise stated. In the case of qualitative variables, frequencies and percentage were obtained. The statistical analysis was performed with IBM SPSS Statistics 20.0 (SPSS, Inc., Armonk, NY).

## 3. Results

### 3.1. Study Population

Table 1 shows the baseline characteristics of all 48 study participants. Almost two-thirds were male (60.4%) with a mean of 38.5 ± SD 12.5 years of age. Almost half of the cases had a past medical history of type 2 diabetes (43.8%). There were no cases with positive HIV infection. The mean body mass index (BMI) of the study group was within normal limits (23.6 ± SD 5.9 kg/m^2^); nevertheless, 30% percent had a low BMI (≤18.5). The mean time elapsed between TB diagnosis and baseline assessment was 29.8 ± SD 17.7 months. Clinical manifestations present in about two-thirds of the participants were myalgias, arthralgias, hyporexia, and weight loss. The mean weight loss was 9.3 ± SD 8.2 kg. There were 23 cases (47.6%) of primary MDR-TB.

### 3.2. Cortisol Responses to Low-Dose ACTH Stimulation Test

The results of the adrenocortical functional reserve before and after stimulation with the low-dose ACTH stimulation test are shown in Table 2. The mean baseline serum cortisol value was 464.1 ± 211.2 nmol/L. The mean percentage increment in serum cortisol at 30 and 60 minutes after stimulation with low-dose ACTH stimulation was 102.7% ± 99.3% and 116.3% ± 115.1%, respectively. Taking 500 nmol/L as the minimally normal serum cortisol response after ACTH stimulation, there were two out of 48 subjects with insufficient cortisol responses (4.2%). The maximum serum cortisol values achieved in these cases at 30 or 60 minutes were 492.2 and 482.8 nmol/L. Taking 550 nmol/L as an alternative cutoff point value, there were four tests with abnormally low serum cortisol levels (8.3%). Basal ACTH values in these cases were 80 pg/mL, 52 pg/dL, 42 pg/dL, and 46 pg/dL, respectively (Table 3). Adrenocortical function was assessed 10.2 ± SD 3.6 months after initiation of therapy after TB intensive treatment phase and once culture was negative. At follow-up, all patients with a baseline insufficient adrenocortical response returned to normal values.

### 3.3. Baseline Clinical Characteristics of the Abnormal Responders

At baseline, there were two and four cases with insufficient cortisol responses (≤500 nmol/L and ≤550 nmol/L, resp.). The mean time from TB diagnosis to the assessment in these cases was 37.5 ± 15.8 months. Besides weakness, the three most common clinical features in these subjects were weight loss, arthralgia, and myalgia. Three out of four had secondary MDR-TB and 50% had diabetes as comorbidity. They previously had been on the antituberculosis medications, Rifampicin, Isoniazid, Ethambutol, and Pyrazinamide, and were taking Amikacin, Levofloxacin, Prothionamide, Cycloserine, Pyrazinamide, and Ethambutol at the time of this study.

## 4. Discussion

In this prospective study of patients with MDR-TB, (half of them with primary drug-resistance and type 2 diabetes, without HIV infection) using the low-dose ACTH stimulation test, we found two cases (4.2%) of adrenal insufficiency using the 500 nmol/L cutoff cortisol value and 4 cases (8.3%) using the 550 nmol/L criterion. Interestingly, at the 10.2 ± SD 3.6-month follow-up, once mycobacterial cultures became negative, the adrenal response to the low-dose ACTH stimulation test was restored in all four cases. These findings have important clinical implications as this study represents to our knowledge the first to recognize the behavior and response of the adrenocortical function, using the low-dose ACTH stimulation test, in MDR-TB HIV-negative patients after intensive antituberculosis treatment.

The relationship between adrenal dysfunction and tuberculosis has been known for a long time and it is still mentioned in some textbooks as a common etiology of adrenal insufficiency [5]. Nevertheless, the prevalence of adrenal insufficiency secondary to tuberculosis has been reported to be in a wide range [8–11]. Moreover, in some case series over 70% of the cases attributed to tuberculosis have been diagnosed after an autopsy [16]. In 1930, Guttman reported that tuberculosis was found in the adrenal glands in 69.7% of the 566 patients that were previously known to have Addison's disease. This was a postmortem study and no adrenocortical stimulation test was realized [17]. In 1986, in South Africa, 55% of patients with active tuberculosis were found to have an insufficient response to the high-dose ACTH stimulation test; there was no follow-up and the extent of the tuberculosis infection in the cases was not clearly described [18]. Later on, Barnes et al. in a pioneer study that involved patients with active tuberculosis (30 pulmonary, 30 miliary, and 30 extrapulmonary) evaluated the adrenocortical function before and three to four weeks after antituberculosis treatment. No patient had a low basal cortisol level and all except for one patient had a normal response to the high-dose ACTH stimulation test [19]. Also, Chan et al. in 39 patients with active pulmonary tuberculosis reported that 41% had an insufficient adrenocortical response to the high-dose ACTH stimulation test. Basal cortisol concentrations were found to be higher between survivors than nonsurvivors and after antituberculosis treatment only 10% of the patients remained having a subnormal cortisol response [20]. Unfortunately, in all of these previous studies, the criteria to assess the adrenal function were different to what we use nowadays; the definition used as an adequate response was a cortisol increase of ≥200 nmol/L from baseline 30 minutes after the high-dose ACTH stimulation test. More recently, 49.5% of 97 HIV-negative patients with various forms of tuberculosis were found to have an insufficient cortisol response to the high-dose ACTH stimulation test. Only 13 patients were followed up and in nine subjects the adrenocortical response returned to normal after antituberculosis treatment [11]. This is a considerably higher prevalence that the one found in our study, different disease duration and/or therapies, may have played a role. Using the low-dose ACTH stimulation test, Kaplan et al. found that only 2.5% of hospitalized patients with active pulmonary tuberculosis had an insufficient adrenocortical response. Forty-five percent of the patients were HIV-positive; all of them were newly diagnosed and no follow-up was made. Interestingly, the mean cortisol after low-dose ACTH stimulation did not differ significantly when comparing either patients and controls or patients who were HIV-positive and HIV-negative [21]. Finally, Odeniyi et al. in 44 patients with newly diagnosed pulmonary tuberculosis, using the low-dose ACTH stimulation test, documented that 27% of them had an insufficient cortisol response; no follow-up was made [8]. To our knowledge, the prevalence of adrenocortical failure has never been described before in MDR-TB HIV-negative patients and using the low-dose ACTH stimulation test. Furthermore, the adrenocortical response to a low-dose ACTH stimulation test was assessed before and after a high-rate compliance of antituberculosis treatment. Less than 10% of the cases had insufficient cortisol responses at baseline. After a negative tuberculosis culture, all cases had a normal cortisol response on stimulation. It is important to remark that none of the four patients with adrenal insufficiency were receiving Rifampicin (associated as a potential cause of adrenal insufficiency by increasing cortisol clearance (enzyme-inducing effects)) at the time of our assessment. In addition, ten patients with secondary drug-resistance were using it at baseline and all of them had a normal cortisol response to the low-dose ACTH stimulation test.

Several theories behind the pathophysiology between adrenal insufficiency and tuberculosis have been proposed and it has been considered that patients with a long-standing, miliary or with poor compliance to tuberculosis therapy are more prone to have adrenal dysfunction compared to those with a newly diagnosed pulmonary tuberculosis that is rapidly and effectively treated [10, 11, 16, 17, 19, 22]. Consequently, it was easy to hypothesize that, in MDR-TB cases, in which tuberculosis is usually more aggressive, treatment is less effective or given for a longer time or, in the case of a secondary drug-resistance, after a long-lasting irregular and unsuccessful first-line drug-treatment, patients would probably have a higher prevalence of adrenocortical dysfunction and would be less likely to achieve a normal cortisol response after antituberculosis treatment. To our surprise, we found a strikingly low prevalence of adrenal insufficiency. Moreover, in those few cases, after six months of antituberculosis treatment, the adrenocortical cortisol response completely recovered in all cases. The procurement of negative cultures at follow-up before the low-dose ACTH stimulation test, the high-rate compliance of the antituberculosis treatment, and the exclusion of HIV-positive patients are reasons that could explain this phenomenon. It is also important to mention that two cases with adrenal insufficiency (using the 500 nmol/L cortisol cutoff value) had secondary drug resistance. When the 550 nmol/L criterion was used, of the four cases with an insufficient adrenocortical response, one had primary drug-resistance and three had secondary drug-resistance. This was expected, as exposure time and the extent of the disease were higher in the latter (secondary-drug resistance).

HIV infection has been related to HPA axis dysfunction primarily due to opportunistic infections (i.e., patients with AIDS and a very low CD4 cell count) [23–25]. In addition, HIV itself and treatment with protease inhibitors drugs have been related to adrenal insufficiency [26]. In a previous study in our laboratory, in 104 HIV-positive patients, using the low-dose ACTH stimulation test, adrenocortical dysfunction was found in 21% of cases [27]. This has been also documented by other series in cases with HIV infection; however, many of the previous studies that have searched for the association of adrenal insufficiency and tuberculosis have included HIV-positive patients. Hence, our study (which excluded HIV-positive patients) represents a probably more accurate estimation of the real association between tuberculosis and adrenocortical dysfunction.

Several protocols have been used to assess the adrenocortical response to synthetic ACTH. Until recently, the high-dose ACTH stimulation test was the best known and most used test in many centers. Recent studies, however, have documented that 250 *μ*g of ACTH is considered a supraphysiologic stimulus rather than a physiologic stimulus and may result in a normal cortisol response in the setting of mildly dysfunctional adrenocortical glands [12, 14]. This is especially important when a chronic partial pituitary ACTH deficiency or recent onset ACTH deficiency is suspected. In these scenarios, the low-dose ACTH stimulation test has yielded better performance [12, 14, 28, 29]. A recent systematic review by Ospina et al. suggests both tests have similar accuracy. Unfortunately, for primary adrenal insufficiency, they were only able to include 5 studies (enrolling just 100 patients). In consequence, with the available data they were only able to estimate the sensitivity of high-dose ACTH stimulation test (92%; 95% CI: 81%–97%) and no data was provided with regard to the low-dose ACTH test [30]. Hence, this is also a strong point of our study, since tuberculosis is believed to affect the adrenal glands in a progressive and gradual manner. In fact, the four patients with insufficient response to low-dose ACTH stimulation test had a near-normal/partially insufficient cortisol response that could have been otherwise overestimated if we had used the high-dose ACTH stimulation test.

## 5. Conclusions

In patients with MDR-TB we found, using the low-dose ACTH stimulation test, a 5–10% prevalence of adrenal insufficiency. At follow-up, after the intensive phase of antituberculosis treatment, the cortisol response was restored in all cases. The exclusion of HIV-positive patients could explain, at least partially, the low prevalence of an abnormal cortisol response found in our study, representing a more accurate estimation of the real association between tuberculosis and adrenocortical dysfunction. Given the increasing epidemic of TB and rampant surge of MDR-TB these findings have important clinical implications that may help clinicians and patients make better decisions when deciding to test for adrenocortical dysfunction or treat mild stimulated abnormal cortisol levels in the setting of MDR-TB.

## Figures and Tables

**Table 1:** Baseline characteristics of the studied population (*n* = 48).

Age (years)	38.5 ± 12.5
18–35 (*n*/%)	20 (41.7)
36–45 (*n*/%)	12 (25)
>45 (*n*/%)	16 (33.3)
Gender	
Males (*n*/%)	29 (60.4)
Body mass index (kg/m^2^)	23.6 ± 5.9
Clinical manifestations	
Weakness (*n*/%)	27 (64.3)
Hyporexia (*n*/%)	22 (45.8)
Weight loss (*n*/%)	37 (77.1)
<5 kg	5 (13.5)
5–10 kg	16 (43.2)
>10 kg	16 (43.2)
Mean weight loss (kg)	9.3 ± 8.2
Hyperpigmentation (*n*/%)	10 (20.8)
Time of evolution prior to diagnosis (months)	29.8 ± 17.7
Primary MDR-TB (*n*/%)	23 (47.6)
Secondary MDR-TB (*n*/%)	25 (52.4)

MDR-TB: multidrug-resistant tuberculosis.

**Table 2:** Serum cortisol responses to the low-dose ACTH stimulation test at baseline and follow-up.

	Total population (*n* = 48)		Primary MDR-TB (*n* = 23)		Secondary MDR-TB (*n* = 25)	
	Baseline	Follow-up	Baseline	Follow-up	Baseline	Follow-up
Serum cortisol (nmol/L)						
Baseline	464.1 ± 211.2	476.8 ± 306.6	480.1 ± 202.4	467.9 ± 315.6	449.5 ± 222.2	488.7 ± 307.7
30 min	787.2 ± 173.1	912.8 ± 213.8	787.7 ± 179.4	856.6 ± 239.3	786.8 ± 170.8	987.6 ± 152.9
60 min	831.3 ± 192.7	991.8 ± 236.3	854.59 ± 224.9	918.9 ± 265.9	809.9 ± 159.4	1088.9 ± 150.1
Increase in serum cortisol (%)						
0′–30 min	102.7 ± 99.3	160.3 ± 174.9	99.5 ± 120.2	145 ± 163.5	105.7 ± 77.8	180.7 ± 194.5
0′–60 min	116.3 ± 115.1	182.8 ± 188.3	115 ± 135.1	168.1 ± 197.4	117.5 ± 96.1	202.3 ± 182.1
Normal cortisol responders (*n*/%)						
500 nmol/L cutoff point	46, (95.8)	33, (100)	22 (95.6)	19, (100)	24, (96)	14 (100)
550 nmol/L cutoff point	44, (91.7)	33, (100)	22 (95.6)	19, (100)	22, (88)	14 (100)

MDR-TB: multidrug-resistant tuberculosis.

**Table 3:** Basal and peak cortisol levels.

Patient	Basal cortisol (nmol/L)	Peak cortisol (nmol/L)
1	309.28	970.34
2	867.15	769.76
3	212.71	753.2
4	413.57	753.2
5	441.9	839.56
6	673.74	943.3
7	834.59	1086.49
8	229.2	813.9
9	254.93	769.76
10	205.26	835.97
11	597.59	701.88
12	412.19	738.58
13	331.9	800.11
14	462.96	1112.15
15	258.51	580.21
16	739.68	948.54
17	853.35	1211.47
18	307.35	753.48
19	354.53	851.97
20	307.9	816.66
21	742.44	852.25
22	534.41	810.31
23	784.65	810.31
24	919.02	1229.68
25	434.81	1084.56
26	545.73	1006.75
27	62.62	482.82^*∗*^
28	432.61	699.4
29	382.67	829.9
30	656.64	951.02
31	239.75	963.99
32	548.76	737.48
33	567.8	1112.15
34	272.03	523.12^+^
35	380.74	836.52
36	733.61	958.2
37	449.71	859.42
38	686.99	1165.67
39	309	541.29^+^
40	512.62	1236.03
41	382.67	915.98
42	218.23	822.73
43	206.92	697.47
44	493.58	892.53
45	384.6	879.56
46	724.51	855.56
47	320.87	884.81
48	282.79	492.2^*∗*^

^*∗*^Peak cortisol less than 500 nmol/L.

^+^Peak cortisol less than 550 nmol/L.
